# Hemolysis correction factor in the reporting of serum neuron-specific enolase – Clinical utility in neuroprognostication after cardiac arrest

**DOI:** 10.1016/j.resplu.2025.101208

**Published:** 2025-12-24

**Authors:** Christina Jungar, Erik Alinder, Charlotte Becker, Marion Moseby-Knappe, Anna Lybeck

**Affiliations:** a Department of Clinical Chemistry and Pharmacology, University and Regional Laboratories, Region Skåne, Sweden; b Department of Clinical Sciences Lund, Neurology and Rehabilitation, Skåne University Hospital, Lund University, Lund, Sweden; c Department of Clinical Sciences, Anaesthesia and Intensive Care, Skåne University Hospital, Lund University, Lund, Sweden

**Keywords:** Neuron specific enolase, Hemolysis, Cardiac arrest, Neuroprognostication, Biomarkers

## Abstract

**Background:**

Neuron-specific enolase (NSE) from 48 h after cardiac arrest is the only biomarker of brain injury with recommended cut-offs for use in neuroprognostication. Hemolysis elevates levels of NSE and may result in false outcome predictions.

**Methods:**

A correction-factor for hemolysis in reporting of levels of NSE was established and evaluated in (1) incoming routine samples and (2) biobank samples from 48 h after cardiac arrest from the SweCrit biobank. Comparisons were made with three methods for handling hemolysis: Hemolysis Index (HI) 30 mg/dL or HI 50 mg/dL as the highest acceptable level of hemolysis, or a graded approach.

**Results:**

Five-hundred and fifty-six routine samples and 263 biobank samples were analyzed. A correction factor of 0.33 µg/L per HI significantly increased the number of reported routine samples, when compared to the three other methods for handling hemolysis (HI 30 mg/dL or HI 50 mg/dL as the highest acceptable level of hemolysis, or a graded approach). Use of the correction factor did not affect the number of reported biobank samples. The prognostic accuracy of NSE was unaffected by use of the correction factor compared to the other tested methods for handling hemolysis: area under the curve (AUC) 0.88 (95 % Cl 0.84–0.92) vs 0.87 (95 % Cl 0.83–0.92) at HI ≤ 30 mg/dL, 0.87 (95 % Cl 0.83–0.92) at HI ≤ 50 mg/dL and 0.87 (95 % CI 0.83–0.92) with the graded approach. Levels of hemolysis were low in the biobank samples.

**Conclusion:**

Due to the low levels of hemolysis in the biobank samples, the effects of a correction factor on neuroprognostication after cardiac arrest in routine samples remains uncertain. Clinical use of a correction factor may lead to more reported samples but risks over-correction.

## Introduction

Levels of neuron-specific enolase (NSE) in blood are elevated after brain injury and NSE is the only biomarker in blood with recommended cut-offs for clinical use in multimodal neuroprognostication of patients who remain unconscious after cardiac arrest.^1^

**Fig. 1:**
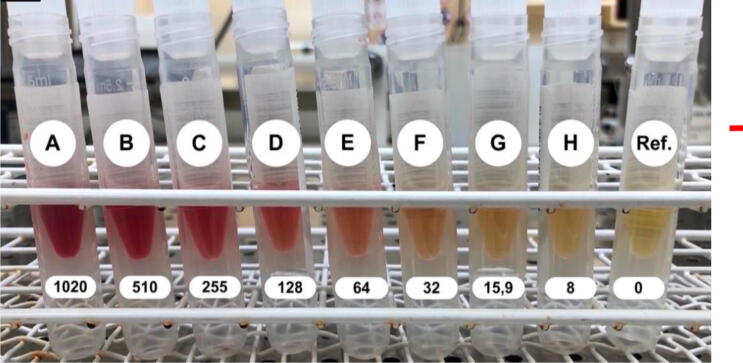
**Serum samples from the dilution series showing hemolysis at HI 0–1020 mg/dL. Visible hemolysis is generally considered to be present at HI 30 mg/dL (depending on the operator and environmental conditions). A cut-off at HI50 mg/dL is common for reporting a measured the NSE**.

Measurement of NSE levels in blood samples (most commonly serum) can be complicated by the high concentration of NSE in erythrocytes and its presence in cells of neuroendocrine origin.^2^ Both in vitro and in vivo hemolysis render elevated measured NSE-concentrations. The concentration of free hemoglobin in samples is described by the hemolysis index (HI). Laboratories commonly choose not to report NSE for samples with a HI > 50 mg/dL or with visible hemolysis, which occurs at about HI 30 mg/dL depending on the operator and environmental conditions (Fig. 1).^8^

Laboratories reporting elevated NSE levels due to hemolysis could lead to false pessimistic predictions of a likely poor outcome in neuroprognostic assessments. On the other hand, being overly cautious with hemolytic samples limits the clinical availability of NSE results.

A number of studies have investigated a correction factor for reporting NSE based on the degree of hemolysis measured in samples, automated algorithms for routine clinical reporting have been suggested but none have evaluated the clinical utility of a correction factor after cardiac arrest.^3^, ^4^, ^5^, ^6^, ^7^, ^8^

The aim of the present study was to establish a correction factor for hemolysis for the reporting of NSE-levels and assess the clinical utility of the correction factor including effects on neuroprognostication after cardiac arrest. We hypothesized that the use of a correction factor would improve the availability of reported NSE results and improve its prognostic accuracy for neuroprognostication in cardiac arrest patients.

## Materials and methods

### Establishment of a correction factor

Spare EDTA-whole blood from 100 de-identified routine samples were collected at the Clinical Chemistry Laboratory at Skane University Hospital, Sweden between June 2022 and April 2023 and used for the preparation of hemolysates. Hemolysates were prepared according to previous work with slight modifications.^3^ The blood samples were washed with physiological NaCl-solution. Deionized water was added, the samples were vortexed and then frozen at −80 °C for one hour or at −20 °C for 2 h. The samples were thawed, then centrifuged to remove cell debris and poured over into new tubes. Free hemoglobin concentrations were measured on the Sysmex Analyzer XN-10/XN-20 analyzer, Sysmex Corporation Kobe, Japan.

Based on the results of the free hemoglobin measurements, the hemolysates were diluted with a serum pool with low NSE (<17 µg/L) and low HI (<5 mg/dL), to a predefined free hemoglobin concentration of 100 mg/dL, i.e. HI 100 mg/dL. NSE was measured in each sample and the addition of NSE per unit HI was calculated for each sample.

The linearity between NSE and the free hemoglobin concentration was studied by diluting one hemolysate with a free hemoglobin concentration of 1000 mg/dL with the serum pool in steps of 1:2. The final dilution series consisted of eight separate concentration levels with HI between 1020 and 8 mg/dL, where NSE and HI were measured.

### The locally employed graded approach to hemolysis

When reporting NSE-levels, the Clinical Chemistry Laboratory Skane University Hospital Sweden currently uses a graded approach to hemolysis according to NSE-level. NSE levels are reported uncorrected but only for samples where the measured NSE and HI are within the following ranges: NSE (µg/L)/HI (mg/dL) < 17/≤500; 17–22/≤10; 23–40/≤20; 41–60/≤30; 61–100/≤40; 101–400/≤100. The method is based on-in-house studies.

### Routine clinical samples

All results for NSE and HI from de-identified routine samples ordered between March–May 2023 and between January–March 2024 were used as a model to evaluate the number NSE-tests that could be reported by the laboratory services, when using a correcting factor for hemolysis. For logistic reasons a continuous six-month period of data collection could not be performed. For this part of the study ethical review by the Swedish Ethical Review Authority was not required.

### Biobank samples

For clinical validation in outcome prediction after cardiac arrest, we applied the correction factor on biobank serum samples from the SweCrit Biobank (ClinicalTrials.gov no. NCT04974775), including adult (>18 years) cardiac arrest patients admitted to three intensive care units 2014–2018.^9^ Serum samples in the current study were sampled 48 h (±6 h) after cardiac arrest. Sampling procedures were the same as for routine blood samples in the ICU, i.e. usually from an arterial line. All samples were centrifuged, aliquoted, and frozen to −80 °C before storage in the biobank at Region Skane, Sweden (BD-47, SC-1922). Functional outcome according to the Cerebral Performance Category (CPC) scale was assessed at 2–6 months, and dichotomized into good (CPC1–2) and poor outcome (CPC3–5).^10^ Ethical approval was granted by the Regional Ethical Review Board Lund (no. 2014/47) and the Swedish Ethical Review Authority (no. 2022-02681-01). Written informed consent was obtained from patients who regained mental capacity.

### NSE analysis

Serum NSE was measured with a Roche Cobas Pro instrument, Electrochemical Luminiscence-Immuno-assay (ECLIA), Roche Diagnostics, Mannheim Germany. The coefficient of variation was 3.2 % at the level of 11 µg/L and 3.1 % at the level of 90 µg/L. The measuring range was 0.75–300 µg/L, and samples above the measuring range were diluted 1:5 until a numerical value was attained. A NSE of <17 µg/L was considered normal.

### Hemolysis index

HI index was measured with Atellica CH 930, Siemens Healthineers, Siemens Healthcare, Erlangen, Germany. The analytical precision for measurement of HI was evaluated by measuring 12 different HI levels (range 6–161) in 5 replicates.

### Statistics

Clinical baseline data are presented in numbers (percentages) or median and interquartile range (IQR). Normally distributed laboratory results are presented as mean and standard variation (SD), non-normally distributed are presented as median and interquartile range (IQR). Categorical variables are presented as numbers and percentages. Groups were compared using Students *t*-test, Mann–Whitney *U* test or Chi-squared test as appropriate. Linear regression was used to correlate HI and NSE.

Overall diagnostic performance for binary functional outcome was assessed by the area under the receiver operating characteristic curve (AUC). Cutoffs at a set low false positive rate and high sensitivity cut-offs currently used in neuroprognostication are evaluated.^1^, ^11^, ^12^, ^13^ A *p*-value of <0.05 was considered significant. Statistical Analysis was performed using Excel, Microsoft Office Professional Plus, 2013 and Analyze-IT for Microsoft Excel 6.15.4.

## Results

### Establishing a correction factor

When diluting one hemolysate with a serum pool with low levels of hemolysis and NSE, there was an excellent linear relationship between HI and NSE concentrations, *r*^2^ = 0.999 (Supplementary Fig. 1). Coefficient of variation (CV) in measurement of HI at 12 different HI levels in five replicates was 2–8 % in the range of 6–161.

**Fig. 2:**
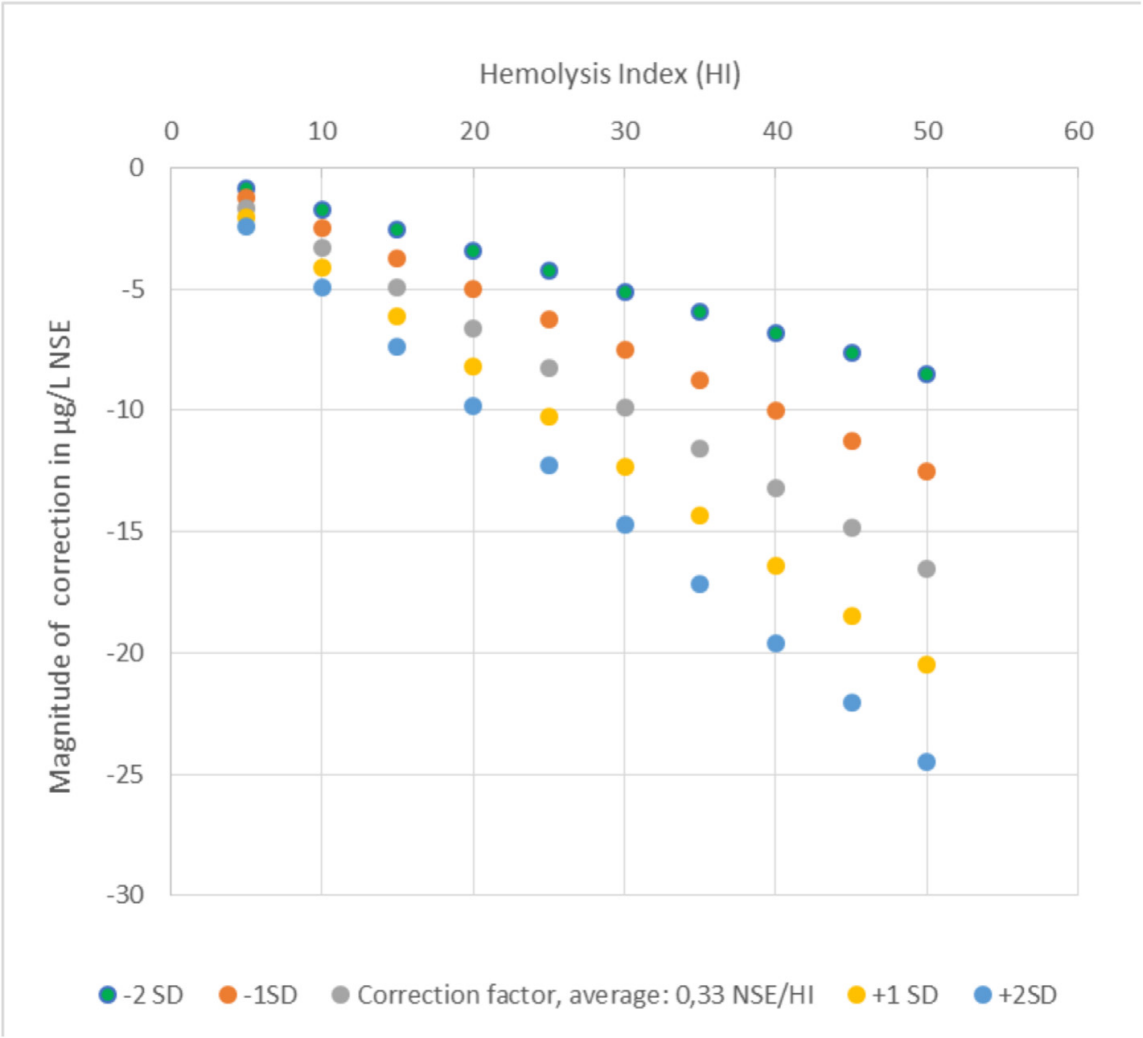
**Incremental effects of a correction factor 0.33 µg/L/, ±1 SD (±0.08 µg/L NSE per HI) and ±2 SD (±0.16 µg/L NSE per HI) when HI in range 0–50 mg/dL**.

In hemolysates from 100 individuals, the mean addition of NSE per HI unit was 0.33 (SD 0.08) µg/L, with an inter-individual CV of 24 % (Supplementary Fig. 2). At HI 30 and HI 50, employing the correction factor 0.33 per HI unit would adjust the reported NSE value by −9 µg/L and −16.6 µg/L respectively. The magnitude of corrections at HI 0–50 including correction at ±1 SD and ±2 SD are visualized in Fig. 2.

### Effects of approaches to hemolysis on number of reported samples

**Fig. 3:**
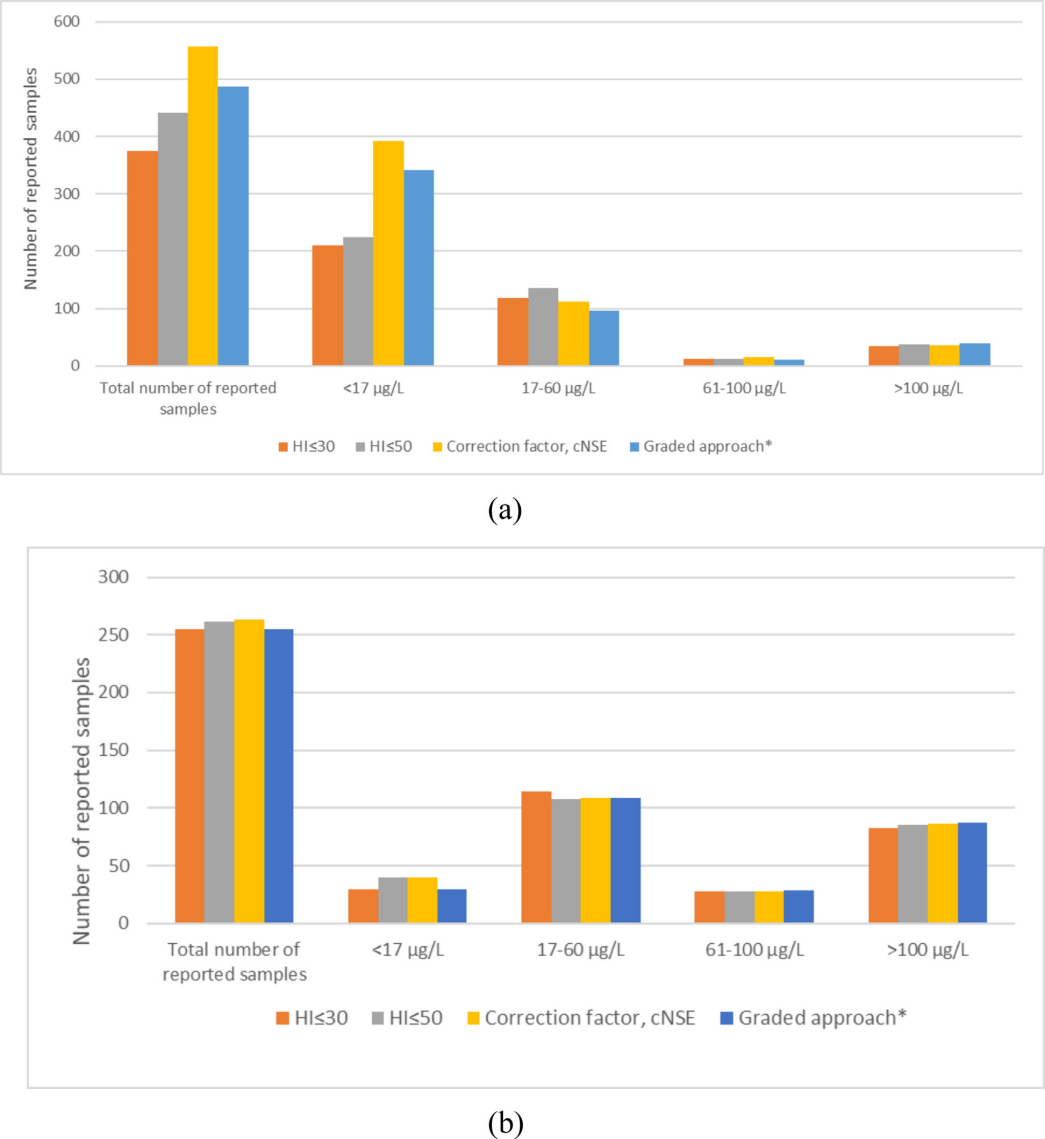
**Number of reported NSE samples when applying different hemolysis rules. NSE < 17 μg/L = normal range, NSE > 60 μg/L = cut-off for prediction of poor outcome after cardiac arrest according to current guidelines, NSE > 100 μg/L = suggested alternative cut-off for safer prediction of poor outcome (11–13)**. (A) Routine samples (*n* = 556). (B) Biobank samples 48 h after cardiac arrest (*n* = 263). *A locally employed graded approach to hemolysis used at the Clinical Chemistry Laboratory Skane University Hospital Sweden according to NSE-level: NSE (µg/L)/H-index (mg/dL): <17/500; 17–22/10; 23–40/20; 41–60/30; 61–100/40; 101–400/100.

During a sample period of 6 months, 556 consecutive incoming routine samples were analyzed for NSE and HI. When employing HI 30 or HI 50 as the highest acceptable level of hemolysis, 67 % (374/556) and 79 % (441/556) respectively of samples would have been reported. With the graded approach currently in use at Clinical Chemistry Laboratory Skane University Hospital Sweden, 88 % (487/556) of the samples would be reported (Fig. 3A). Employing the correction factor on all samples resulted in significantly more reported samples than each of the other 3 approaches (*p* < 0.001).

Serum samples from 263 cardiac arrest patients in the SweCrit cohort were collected at 48 h after cardiac arrest (supplementary Fig. 3, supplementary Table 1). Employing HI 30 as the highest acceptable level of hemolysis, 97 % (256/263) patient samples would be reported. Accepting a higher cut-off HI 50 increased the number of reported NSE samples to 99 % (261/263). With the graded approach to hemolysis currently in use, 97 % (255/263) of the samples would be reported. The distribution of samples reported at NSE levels <17 µg/L, 17–60 µg/L, 61–100 µg/L and <100 µg/L for each strategy for hemolysis including application of the correction factor is reported in Fig. 3B.

The two sets of samples studied differed regarding the levels of measured hemolysis. In the routine samples median HI was 16.7 (IQR 8.1–40.3) and in biobank samples median HI was 2.7 (IQR 1.3–7.4) (*p* < 0.001).

### Effects on the correction factor on outcome prediction after cardiac arrest

**Fig. 4:**
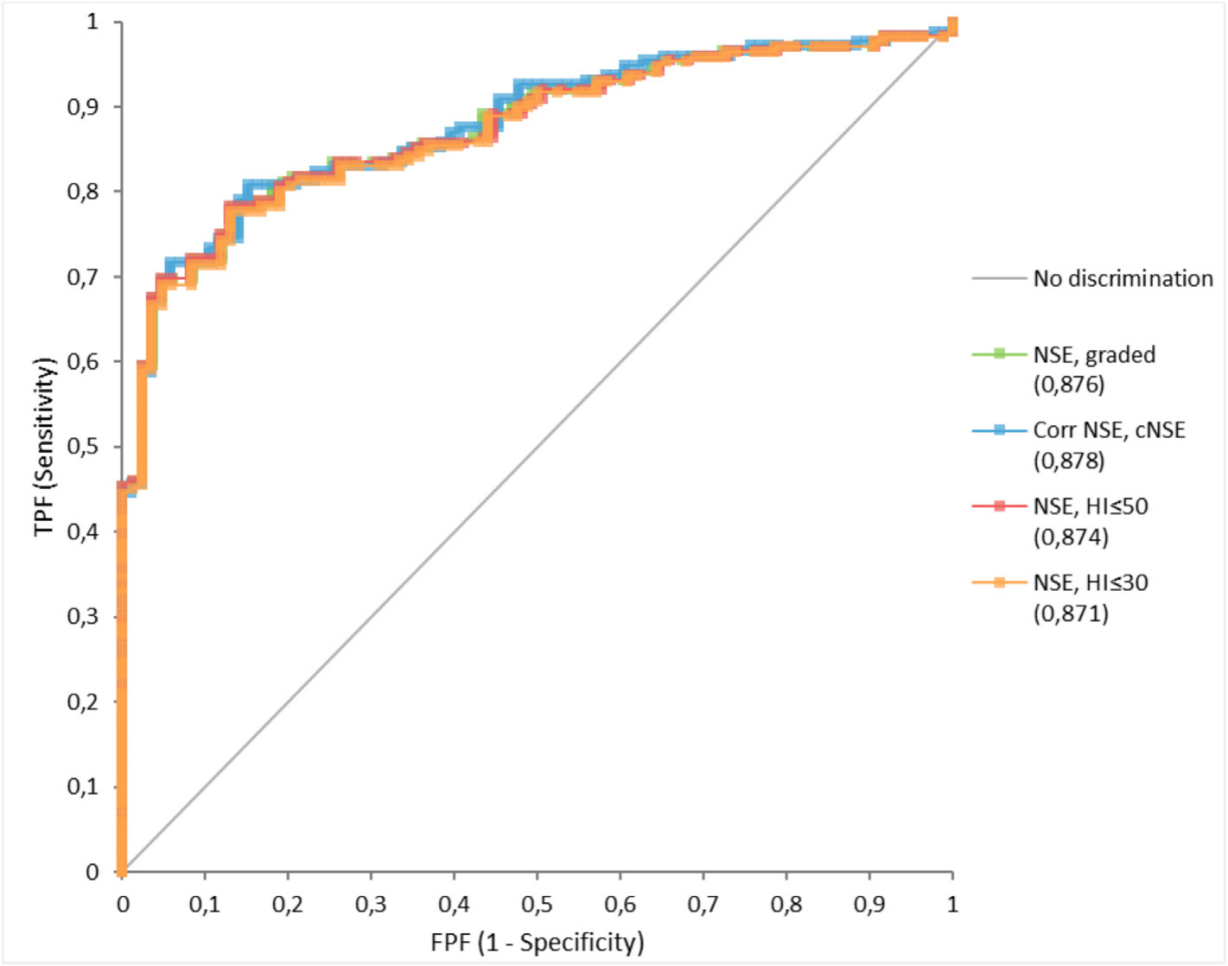
**Receiver Operating Characteristics (ROC) curves with corresponding areas under the curve (AUC) for NSE in predicting poor outcome (CPC 3–5) 48 h after cardiac arrest, when applying four different approaches to hemolysis: graded approach (AUC 0.88 95 %CI 0.84–0.92), the correction factor (AUC 0.88 95 %CI 0.84–0.92), HI ≤ 50 (AUC 0.87 95 %CI 0.83–0.92), and HI ≤ 30 (AUC 0.87 95 %CI 0.83–0.92)**. *A locally employed graded approach used at the Clinical Chemistry Laboratory Skane University Hospital Sweden to hemolysis when reporting NSE according to NSE-level: NSE (µg/L)/H-index (mg/dL): <17/500; 17–22/10; 23–40/20; 41–60/30; 61–100/40; 101–400/100.

The AUCs of NSE at 48 h after cardiac arrest as a predictor of poor neurological outcome using the four approaches to hemolysis are shown in Fig. 4. Using the correction factor resulted in an AUC 0.88 (95 % Cl 0.84–0.92). When using HI 30 as the highest acceptable level of hemolysis the AUC of the reported samples was 0.87 (95 % Cl 0.83–0.92). A cut-off of HI 50 resulted AUC 0.87 (95 % Cl 0.83–0.92). With the graded approach, AUC was 0.88 (95 % CI 0.84–0.92). Table 1 reports the ability of NSE at 48 h after cardiac arrest to predict a poor outcome at the recommended cut-offs >60 µg/L and >100 µg/L and a cut-off at high specificity (FPR  2 %) using the four different approaches to hemolysis. The prognostic accuracy of a normal NSE (<17 µg/L) to predict a good outcome when using the four different approaches to hemolysis (use of the correction factor, HI 30 mg/dL or HI 50 mg/dL as the highest acceptable level of hemolysis, or a graded approach) is shown in Table 2. Prediction of both poor and good outcome was largely unaffected by approach to hemolysis, although sensitivity for prediction of a good outcome may be improved with the use of a correction factor.

**Table 1:** Prediction of a poor outcome (CPC3–5) at high specificity (FPR ≤ 2 %) by NSE at 48 h ours after cardiac arrest at cut-offs >60 µg/L and >100 µg/L using the four different approaches to hemolysis.

**Approach to hemolysis**	**NSE Cut off** **(µg/L)**	**FPR % (95 %CI)**	**Sensitivity % (95 %CI)**	**True positive**	**False positive**	**True negative**	**False negative**	**Total**
Correction factor 0.33 µg/L per HI	>60	3.5 (1.2–9.8)	62.1 (54.8–69.0)	110	3	83	67	263
	>100	2.3 (0.6–8.2)	45.8 (39.0–53.1)	83	2	84	94	263
	105	<2% Actual FPR 1.2 (0–3.6)	45.8 (38.4–53.1)	81	1	85	96	263

HI ≤ 30	>60	3.6 (1.2–9.9)	62.6 (55.1–69.5)	107	3	81	64	255
	>100	2.4 (0.7–8.3)	46.8 (39.5–54.2)	80	2	82	91	255
	105	<2% Actual FPR 1.3 (0.2–6.9)	45.0 (37.8–52.5)	77	1	83	94	255

HI ≤ 50	>60	3.5 (1.2–9.9)	63.6 (56.3–70.4)	112	3	82	64	261
	>100	2.4 (0.6–8.2)	47.7 (40.5–55.1)	84	2	83	92	261
	105	<2% Actual FPR 1.2 (0.2–6.6)	46.0 (38.7–53.4)	81	1	84	95	261

Graded approach*	>60	3.7 (1.3–10.3)	64.4 (57.0–71.1)	112	3	78	62	255
	>100	2.4 (0.7–8.6)	48.3 (41.0–55.7)	84	2	79	90	255
	105	<2% Actual FPR 1.2 (0–3.6)	46.6 (39.1–54.0)	81	1	80	93	255

CI = confidence interval; CPC = Cerebral Performance Category; FPR = false positive rate; HI = Hemolysis Index; NSE = neuron-specific enolase.

* A locally employed graded approach to hemolysis when reporting NSE according to NSE-level: NSE (µg/L)/H-index (mg/dL): <17/500; 17–22/10; 23–40/20; 41–60/30; 61–100/40; 101–400/100.

**Table 2:** The ability of a normal NSE (<17 µg/L) to predict a good outcome when using the four different approaches to hemolysis. The majority of patients dying despite normal NSE levels reportedly had a physician reported non-neurological presumed cause of death.

**Approach of hemolysis**	**FPR (%)**	**Sensitivity (%)**	**Negative predictive value**	**Positive predictive value**	**True positive**	**False positive**	**True negative**	**False negative**	**Total**
Correction factor 0.33 µg/L per HI	4.5 (2.3–8.7)	37.2 (27.8–47.4)	75.6 (69.7–80.1)	20.0 (10.5–34.8)	32	8	169	54	263
HI ≤ 30	4.1 (2.0–8.2)	27.4 (19.0–37.8)	73.3 (67.2–78.7)	23.0 (11.8–40.9)	23	7	164	61	255
HI ≤ 50	4.0 (1.9–8.0)	27.1 (18.8–37.4)	73.1 (67.1–78.5)	23.0 (11.8–40.9)	23	7	169	62	261
Graded approach*	4.0 (2.0–8.1)	28.4 (19.7–39.0)	73.1 (67.1–78.5)	23.0 (11.8–40.9)	23	7	167	58	255

CI = confidence interval; CPC = Cerebral Performance Category; FPR = false positive rate; HI = hemolysis index; NSE = neuron-specific enolase.

* A locally employed graded approach to hemolysis when reporting NSE according to NSE-level: NSE (µg/L)/H-index (mg/dL): <17/500; 17–22/10; 23–40/20; 41–60/30; 61–100/40; 101–400/100.

## Discussion

In this study, we established a factor of 0.33 µg/L per HI unit for correcting reported NSE-values for detected hemolysis and compared it with three methods currently in use. Applying the correction factor markedly increased the proportion of reported routine samples. Among biobank samples from 48 h after cardiac arrest, both the proportion of reported samples and the ability of NSE to predict outcome was largely unaffected by the use of the correction factor compared the other three investigated approaches to hemolysis (HI 30 mg/dL or HI 50 mg/dL as the highest acceptable level of hemolysis, or a graded approach). This discrepancy may be explained by the lower levels of detected hemolysis among the biobank samples.

The correction factor 0.33 µg/L per HI unit developed for the present study was similar to that reported by previous investigations, suggesting generalizability of the correction.^3^, ^5^, ^6^, ^7^, ^8^, ^14^ As expected, the proportion of reported routine samples increased with the use of the correction factor. The use of a correction factor would avoid repeat sampling, benefiting in particular patients with difficult phlebotomy where hemolysis is common including neonatal patients.

Interestingly, the biobank samples from 48 h after cardiac arrest exhibited lower levels of detected hemolysis than the routine samples and the proportion of biobank samples with reported NSE did not increase with the use of the correction factor. Due to the anonymization of the routine samples, information on the ward of origin was not available. We can only speculate that differences in sampling procedures between the ICU and general wards may have affected the degree of hemolysis. Alternatively, ex vivo hemolysis in the biobank samples may have been arrested by freezing.

In the present study the coefficient of variation of the correction factor was 24 %. The concentration of NSE in red blood cells varies up to two-fold between individuals and may limit generalizability of a correction coefficient.^4^ Due to this individual variation, concerns have been raised regarding over-correction when employing a generalized correction factor. Over-correction may e.g. prolong futile intensive care in patients with no chance of recovery but also affect treatment of cancers, in particular small cell lung cancer. Various approaches to avoid over-correction have been suggested. One study suggested limiting the use of a correction factor to HI 5–30.^6^ Another recent study on NSE after cardiac arrest performed correction at HI ≤ 20.^5^ An individualized correction method has been proposed but remains too laborious and costly for laboratories conduct routinely.^7^ A pragmatic approach may be a combination of a lower correction-factor employed at a limited range of HI.

To improve the interpretation of reported NSE samples inclusion of a comment in the laboratory information system may be helpful, e.g. reporting a range of possible corrected NSE-values based on a correction factor ±1 SD and ±2 SD. Improved knowledge about the range of rise in NSE per hemolysis unit could help guide clinical decision making. However, striking a balance between information and “alert-fatigue” among clinicians remains a challenge.

Regardless of what approach to detected hemolysis is employed, the issue of undetected hemolysis affecting measured NSE-levels remains. In vivo the half-life of NSE is 30 h, but the half- life of hemoglobin is only 2–4 h, i.e. a NSE from hemolysed red blood cells may well be measured long after the released hemoglobin is cleared.^15^, ^16^ Additionally, although measured hemolysis is not affected by laboratory method, the measured NSE level does differ between laboratory methods and variation must also be considered when interpreting results.^17^, ^18^, ^19^

We have previously published a validation study of locally reported NSE values for neuroprognostication after cardiac arrest from the SweCrit patient database.^11^ The previous study included patients with a routine NSE available to clinicians, whereas the present study included those with a biobank sample from 48 h after cardiac arrest, explaining the slight differences in the reported predictive ability of NSE between the two studies. Our previous study suggested a cut-off NSE > 101 µg/L at 48 h after cardiac arrest for safe prediction of a poor outcome.^11^ The present study reports a similar cut-off regardless of which method for avoiding effect of hemolysis is used. However, the levels of hemolysis among the biobank samples were lower than in routine samples, limiting conclusions.

## Strengths and limitations

Strengths of the present study are the heterogeneous cohorts, reflecting clinical reality, allowing for more generalizable results, and the large sample sizes. Additionally, the biobank samples were multicentre and long-term neurological outcome was available. The patients in the biobank were subject to multimodal neuroprognostication according to guidelines including a routine NSE as part clinical care, which induced a risk of self-fulfilling prophecy in the current study.^1^ In the routine samples NSE was measured in a clinical setting while the biobank samples were frozen and stored in biobank and thawed for analysis. NSE analyses were performed on Roche Cobas Pro instrument, Electrochemical Luminiscence-Immuno-assay (ECLIA), and our results may not be applicable to other methods of NSE-analysis.

## Conclusion

A hemolysis correction factor for reported NSE-values may increase the number of reported samples and hence the need for re-sampling, but also risks over-correction with subsequent effects on clinical decision making. The effects of a correction factor on neuroprognostication after cardiac arrest in routine samples remains uncertain. Both detected and undetected hemolysis should be considered when interpreting a reported NSE-value. Educational efforts and alert in the laboratory information system may help in guiding clinicians in interpreting reported NSE-levels.

## CRediT authorship contribution statement

**Christina Jungar:** Writing – review & editing, Writing – original draft, Visualization, Methodology, Investigation, Formal analysis, Data curation, Conceptualization. **Erik Alinder:** Writing – review & editing, Methodology, Formal analysis, Data curation. **Charlotte Becker:** Writing – review & editing, Resources, Methodology, Conceptualization. **Marion Moseby-Knappe:** Writing – review & editing, Methodology, Funding acquisition, Conceptualization. **Anna Lybeck:** Writing – review & editing, Writing – original draft, Supervision, Resources, Project administration, Methodology, Investigation, Funding acquisition, Formal analysis, Data curation, Conceptualization.

## Funding

This work was supported by The Swedish Heart-Lung Foundation [grant number 20240803]; Regional Research Support Region Skane; Skane University Hospital Research Funds; Funding of clinical research within the Swedish National Health Services (ALF); and The Swedish Research Council [grant number 2023-01818].

## Declaration of competing interest

The authors have no conflicts of interest to report.
