# Supplementary material for: Hemolysis correction factor in the reporting of serum neuron-specific enolase – Clinical utility in neuroprognostication after cardiac arrest
Source: Resusc Plus. 2025 Dec 24;28:101208. doi: 10.1016/j.resplu.2025.101208 (PMC12835405; doi:10.1016/j.resplu.2025.101208)
Supplement: Supplementary Data 1 [file mmc1.docx]

**Supplementary Material**

**Supplementary Figure 1:** Correlation between the degree of hemolysis and the rise in NSE concentration (n=1).

**Supplementary Figure 2:** Frequency distribution of the amount of NSE released as a function of HI (n=100). The mean addition of NSE per HI unit was 0.33 (SD 0.08) µg/L.


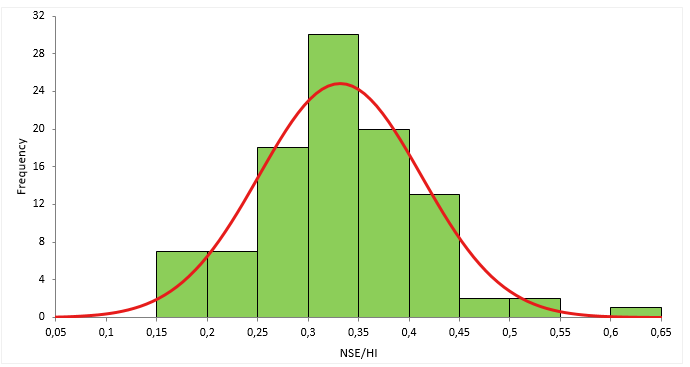


**Supplementary Figure 3** -study flow chart for patients included in the biobank


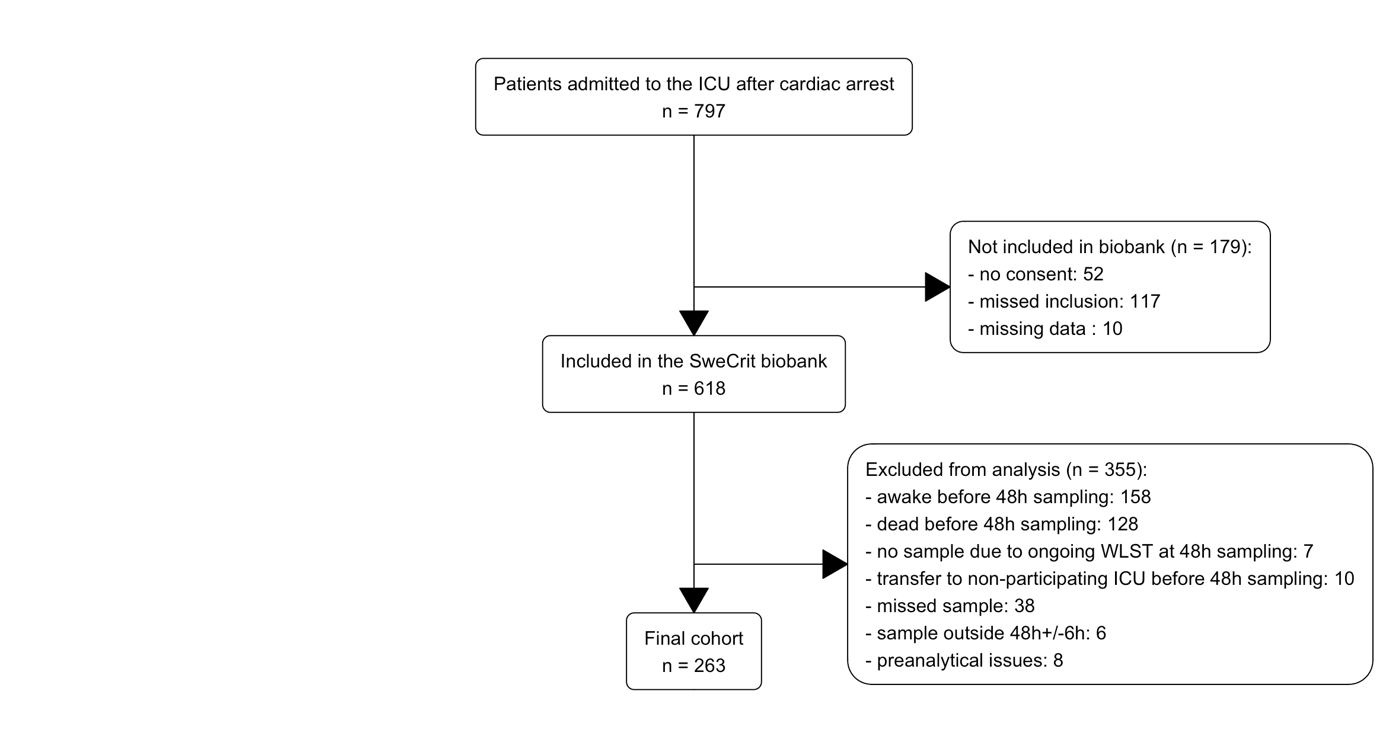


**Supplementary table 1** Characteristics of patients with biobank samples from 48 hours after cardiac arrest

|  | Included (n=263) | Excluded (n=355) |
| --- | --- | --- |
| Age (years) | 67.2 (59.3.5-75.1) | 70.7 (61.2-77.5) |
| Male sex | 196 (74.5%) | 241 (67.9%) |
| OHCA | 209 (79.5%) | 227 (63.9%) |
| Time to ROSC (minutes) | 20 (14-35) | 16 (10-30) |
| Initial rhythm shockable | 122 (46.4%) | 133 (37.5%) |
| Cardiac cause of arrest^a^ | 177 (67.3%) | 207 (58.3%) |
| Withdrawal of life support^b^ | 146 (55.5%) | 126 (35.5%) |
| Time to withdrawal of life support (days) | 3.1 (4-5.9) | 0.7 (1.6-3.8) |
| Poor outcome (CPC3-5) at follow-up | 177 (67.3%) | 221 (62.3%) |

Results are presented as median (interquartile range) or numbers (percentages).

CPC = Cerebral Performance Scale), OHCA=Out-of Hospital Cardiac Arrest; ROSC=return of spontaneous circulation

^a^Retrospectively evaluated during the hospital stay

^b^Multiple reasons for Withdrawal of life support possible including neurological, circulatory, multi-organ failure, comorbidities, or ethical reasons.
